# Photoperiod induced obesity in the Brandt's vole (*Lasiopodomys brandtii*): a model of ‘healthy obesity’?

**DOI:** 10.1242/dmm.026070

**Published:** 2016-11-01

**Authors:** Xin-Yu Liu, Deng-Bao Yang, Yan-Chao Xu, Marianne O. L. Gronning, Fang Zhang, De-Hua Wang, John R. Speakman

**Affiliations:** 1 State Key Laboratory of Molecular Developmental Biology, Institute of Genetics and Developmental Biology, Chinese Academy of Sciences, Beijing 100101, China; 2 Diabetes Research Unit, Novo Nordisk A/S, Novo Nordisk Park, Måløv 2760, Denmark; 3 State Key Laboratory of Integrated Management of Pest Insects and Rodents, Institute of Zoology, Chinese Academy of Sciences, Beijing 100101, China; 4 Institute of Biological and Environmental Sciences, University of Aberdeen, Aberdeen AB24 3FX, UK

**Keywords:** Brandt's vole, Photoperiod, Healthy obesity, Glucose tolerance, Insulin sensitivity, Lipotoxicity, Adipose tissue expandability

## Abstract

Brandt's voles have an annual cycle of body weight and adiposity. These changes can be induced in the laboratory by manipulation of photoperiod. In the present study, male captive-bred Brandt's voles aged 35 days were acclimated to a short day (SD) photoperiod (8L:16D) for 70 days. A subgroup of individuals (*n*=16) were implanted with transmitters to monitor physical activity and body temperature. They were then randomly allocated into long day (LD=16L:8D) (*n*=19, 8 with transmitters) and SD (*n*=18, 8 with transmitters) groups for an additional 70 days. We monitored aspects of energy balance, glucose and insulin tolerance (GTT and ITT), body composition and organ fat content after exposure to the different photoperiods. LD voles increased in weight for 35 days and then re-established stability at a higher level. At the end of the experiment LD-exposed voles had greater white adipose tissue mass than SD voles (*P*=0.003). During weight gain they did not differ in their food intake or digestive efficiency; however, daily energy expenditure was significantly reduced in the LD compared with SD animals (ANCOVA, *P*<0.05) and there was a trend to reduced resting metabolic rate RMR (*P*=0.075). Physical activity levels were unchanged. Despite different levels of fat storage, the GTT and ITT responses of SD and LD voles were not significantly different, and these traits were not correlated to body fatness. Hence, the photoperiod-induced obesity was independent on disruptions to glucose homeostasis, indicating a potential adaptive decoupling of these states in evolutionary time. Fat content in both the liver and muscle showed no significant difference between LD and SD animals. How voles overcome the common negative aspects of fat storage might make them a useful model for understanding the phenomenon of ‘healthy obesity’.

## Abstract (precis)

**Summary:** Obesity induced by long-day photoperiod in voles is not accompanied by impaired glucose tolerance or insulin sensitivity and might be a useful model of ‘healthy obesity’.

## INTRODUCTION

The world is currently faced by two health epidemics. The first is the expansion in levels of obesity, and the second is the increase in levels of type 2 diabetes (Ogden et al., 2006; Scheen, 1999; Schwartz and Porte, 2005). Although obesity and type 2 diabetes are closely linked with one another the association is not inevitable (Scheen, 1999; Schwartz and Porte, 2005; Blüher, 2010). There are many patients who develop obesity but do not develop any metabolic complications. This population has been generally called the ‘healthy obese’ (Blüher, 2010). Understanding why some people are able to become obese without metabolic compromises is important because this might point a way towards novel therapeutic options that will help reduce the translation of obesity into type 2 diabetes. Given the failure of our attempts to solve the obesity problem, stemming the translation from obesity to type 2 diabetes could be a more effective option.

One hypothesis for the phenomenon of ‘healthy obesity’ is the lipotoxicity hypothesis (Virtue and Vidal-Puig, 2008; Unger et al., 2010). The lipotoxicity hypothesis suggests that if an individual builds sufficient fat cells in which ingested and synthesized lipids can be deposited (also called the adipose tissue expandability hypothesis), the individual will largely be protected from the metabolic sequalae of the increased obesity, which are hypothesized to stem mostly from ectopic deposition of fat into the liver and muscle. This idea is supported by several lines of evidence (Virtue and Vidal-Puig, 2008). First, lipodystrophic individuals, who have no white adipose tissue, might be expected, given the broad correlation between increasing obesity and ill health, to be very healthy, but in fact they have severe insulin resistance and metabolic complications (Barroso et al., 1999; Savage et al., 2003). Second, the very existence of the healthy obese population suggests the problem is not obesity per se, but how ingested excess energy is handled and potentially where it is deposited (Blüher, 2010; Karelis et al., 2005). Finally, some treatments for diabetes, such as thiozoladinediones, improve insulin sensitivity but paradoxically at the same time seem to stimulate further fat expansion (Nichols and Gomez-Caminero, 2007).

Although obesity is widely regarded as a maladaptive response to the high levels of energy supply in modern society (Hall et al., 2012; Speakman, 2013), this cannot be the situation in the case of excess adiposity in wild animals. Many wild animals deposit large adipose tissue stores in advance of anticipated periods of food shortage, or in preparation for a period of high demand such as trans-global migration (Martin, 2008; Speakman and O'Rahilly, 2012). Moreover, many other non-hibernating animals, like small rodents (such as voles, hamsters and lemmings) go through annual cycles of fattening that are not obviously linked to periods of migration or low food supply (Steinlechner and Heldmaier, 1982; Ebling and Barrett, 2008; Bartness et al., 2002). The fundamental argument developed here is that over evolutionary time such animals would likely have evolved mechanisms that enable them to avoid the negative aspects of excess energy balance and fat storage. If that is the case, understanding how such animals avoid the negative consequences of obesity might make them valuable tools to explore the phenomenon of ‘healthy obesity’. We suggest that, on the basis of the lipotoxicity/adipose tissue expandability theory, during natural photoperiod-induced obesity these animals might expand their adipose tissue stores in a manner enabling them to avoid lipid overflow and ectopic fat deposition and hence remain in a healthy state.

Voles are small non-hibernating rodents that are common throughout the Palearctic and Nearctic regions. In the wild, voles show a profound annual cycle of body weight (Li and Wang, 2005; Chen et al., 2012) that is driven largely by photoperiod (Król et al., 2005; Król and Speakman, 2007; Zhao and Wang, 2005, 2006). When voles are exposed to long photoperiods they dramatically increase their body weight compared with voles maintained in short photoperiods. This provides us with a powerful tool where, simply by switching the light regime, we can turn a lean vole into an obese vole within the space of about 5 weeks without any change in the diet. This model therefore allows us to explore the consequences of fat storage, divorced from any simultaneous impacts of a diet change. This provides a considerable advantage because most other models of obesity in rodents involve manipulation of the diet to induce obesity. It is difficult to then separate the impacts of the obesity from the impacts of the diet.

The mechanisms, in terms of energy balance, by which voles and other small mammals such as lemmings and hamsters achieve their obese state, following a photoperiod change, seems to be different in different species. We have shown previously that in short-tailed field voles (*Microtus agrestis*) that the long-photoperiod-induced weight increase is driven by an increase in digestive efficiency (Król et al., 2005), whereas in collared lemmings (*Dicrostonyx groenlandicus*) the photoperiod-induced weight increase is achieved by a suppression of resting energy expenditure linked to reduced levels of UCP1 in brown adipose tissue (BAT) (Powell et al., 2002). Previous work in Brandt's voles has shown food intake is not increased in those individuals exposed to LD and gaining weight (Zhao and Wang, 2006), also suggesting an effect rooted in either digestive efficiency changes or suppressed expenditure. Contrasting these effects, LD-induced weight gains in Siberian hamsters (*Phodopus sungorus*) seem to be driven by elevated food intake (Warner et al., 2010).

In the present study we had three objectives. First, we aimed to characterize more closely the photoperiod-induced obesity model in the Brandt's vole, to establish if the change in obesity is accompanied by changes in food intake, resting and daily energy expenditure and physical activity levels. Second, we explored what the consequences of the long-day-induced obese state are for glucose and insulin tolerance, to establish if this is a useful model of ‘healthy obesity’. Finally, we explored the extent to which fat is deposited in the liver and skeletal muscle as the animals become obese to establish if the changes in glucose homeostasis were consistent with the lipotoxicity/adipose tissue expandability hypothesis.

## RESULTS

### Differences in body mass and body composition between LD and SD groups

**Fig. 1.**
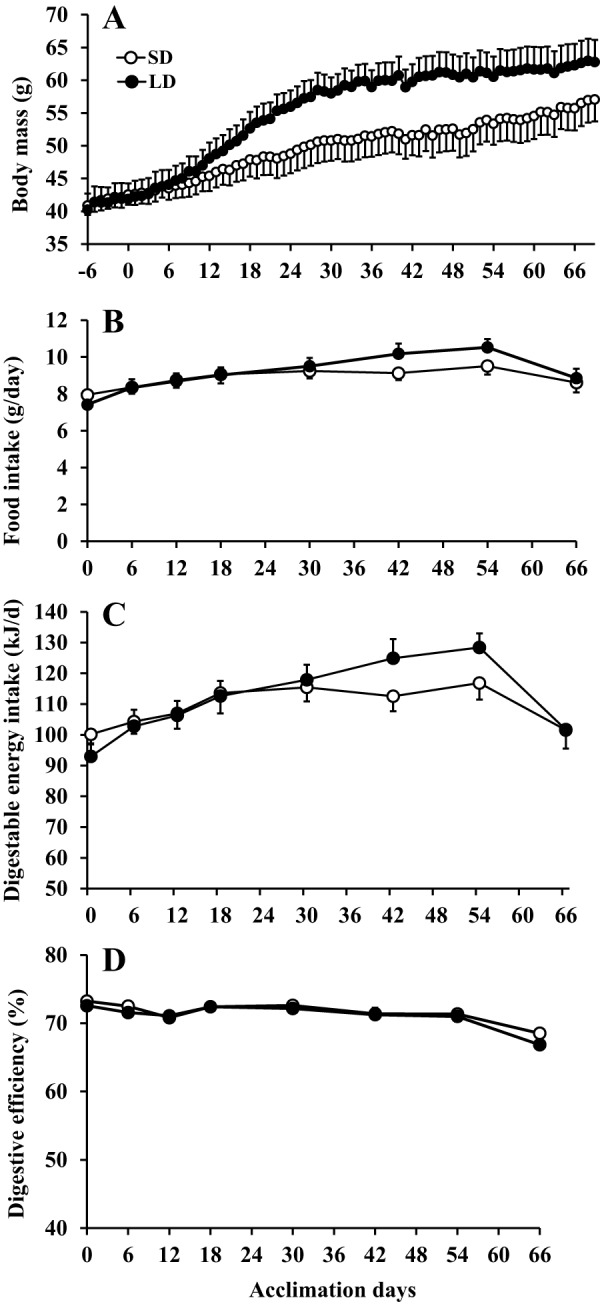
**Effects of photoperiod exposure on body mass and food intake of Brandt's voles.** Thirty-seven voles were exposed to short photoperiod and then 19 of them were switched to a long photoperiod (LD: black points) on day 0, while the remainder (*n*=18) remained on short days (SD: white points). Graphs show (A) body mass, (B) gross food intake, (C) digestible energy intake calculated as the energy in the food minus energy excreted in faeces, and (D) digestive efficiency – the percent of ingested food that is absorbed. Values are means±s.e.m. LD voles gained body weight after the photoperiod switch but this was not associated with elevated food intake, digestible energy intake or altered digestive efficiency.

For the first 6 days after the photoperiod change there was no difference in the body mass between the SD and LD groups (group effect, *F*_1,35_=0.002, *P*=0.969; group×time effect, *F*_6,210_=1.360, *P*=0.232; repeated measures ANOVA, LSD comparison), but thereafter the mass of the LD animals increased and diverged from that of the SD animals and reached maximal levels after 35 days of LD exposure (group effect, *F*_1,35_=1.624, *P*=0.211; group×time effect, *F*_35,1225_=7.140, *P*=0.011; repeated measures ANOVA, LSD comparison; Fig. 1A). From day 35 to day 67 there was no further increase in mass of the LD group (time effect, *P*>0.05). After 70 days of LD exposure, LD voles exhibited significantly greater epididymal, retroperitoneal and inguinal adipose tissue stores, and consequently significantly greater total white adipose tissue (WAT) mass than SD voles (Fig. 1A, Table 1; Fig. S1). Interscapular BAT mass was not significantly different between LD and SD animals (*P*=0.313). LD animals also had significantly larger seminal vesicles than SD voles (Table 1), but other lean tissues including the kidneys, liver, heart, testes and overall fat-free body mass were not significantly different (Table 1).

**Table 1.**
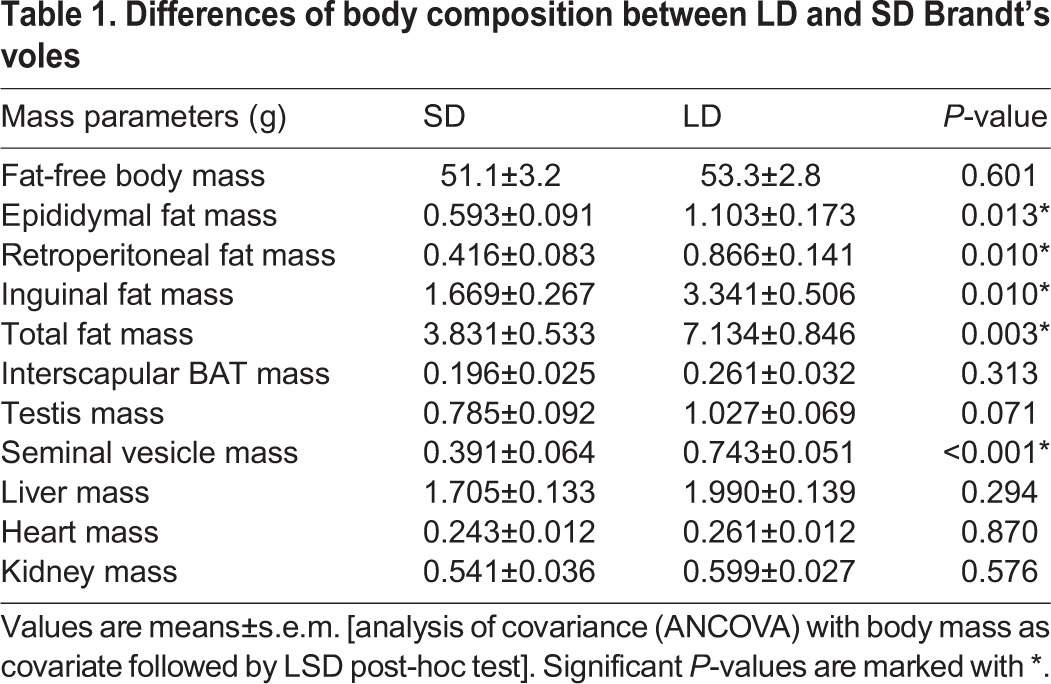
**Differences of body composition between LD and SD Brandt's voles**

### Energy balance

**Fig. 2.**
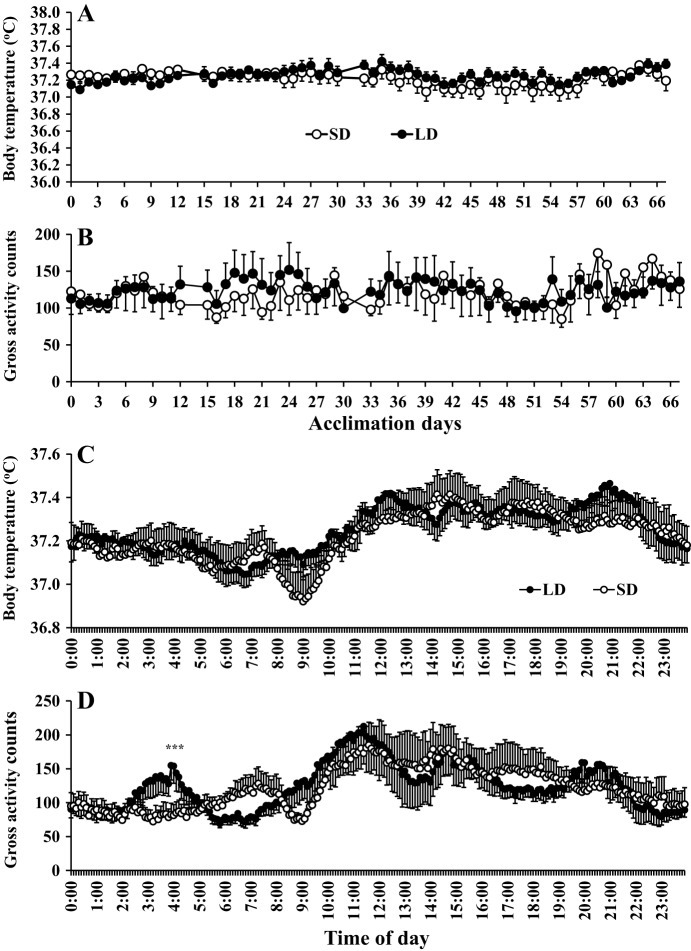
**Effects of photoperiod on aspects of energy expenditure in Brant's voles.** Sixteen voles implanted with transmitters that measure body temperature and physical activity and were exposed to a short photoperiod and then eight of them were switched to a long photoperiod (LD: black points) on day 0, while the remainder (*n*=8) remained on short days (SD: white points). Graphs show (A) daily average body temperature and (B) gross daily physical activity over the 68 days of photoperiod manipulation. (C,D) Twenty-four-hour cycle of body temperature and physical activity of both groups. Voles did not differ in either body temperature or physical activity levels except for a short period as lights came on for the LD animals. ***P<0.05 by repeated-measures ANOVA comparison of LD with SD groups, which was significant on three sequential occasions.

There were no significant differences in daily food intake (Fig. 1B), digestible energy intake (Fig. 1C) and digestive efficiency (Fig. 1D) between the SD and LD groups (ANCOVA using corresponding body mass as covariate, *P*>0.05). For definitions of digestible energy intake (DEI) and digestive efficiency refer to Materials and Methods below. DEI is the amount of energy the animal can extract from the food and the digestive efficiency is the efficiency of that extraction. There were also no significant differences in core body temperature (Fig. 2A) or gross physical activity levels (Fig. 2B) between LD and SD groups throughout the period of photoperiod manipulation (group×time effect, *P*>0.05, repeated measures ANOVA). Diurnal patterns of body temperature (Fig. 2C) and gross physical activity (Fig. 2D) were very similar. The only significant difference was that the LD animals showed transient high activity just prior to their lights coming on at 04:00. No similar pattern was observed in the SD voles when their lights came on. This effect however was small, probably physiologically unimportant and compensated at other times of day as there were no significant differences in overall activity levels.

**Fig. 3.**
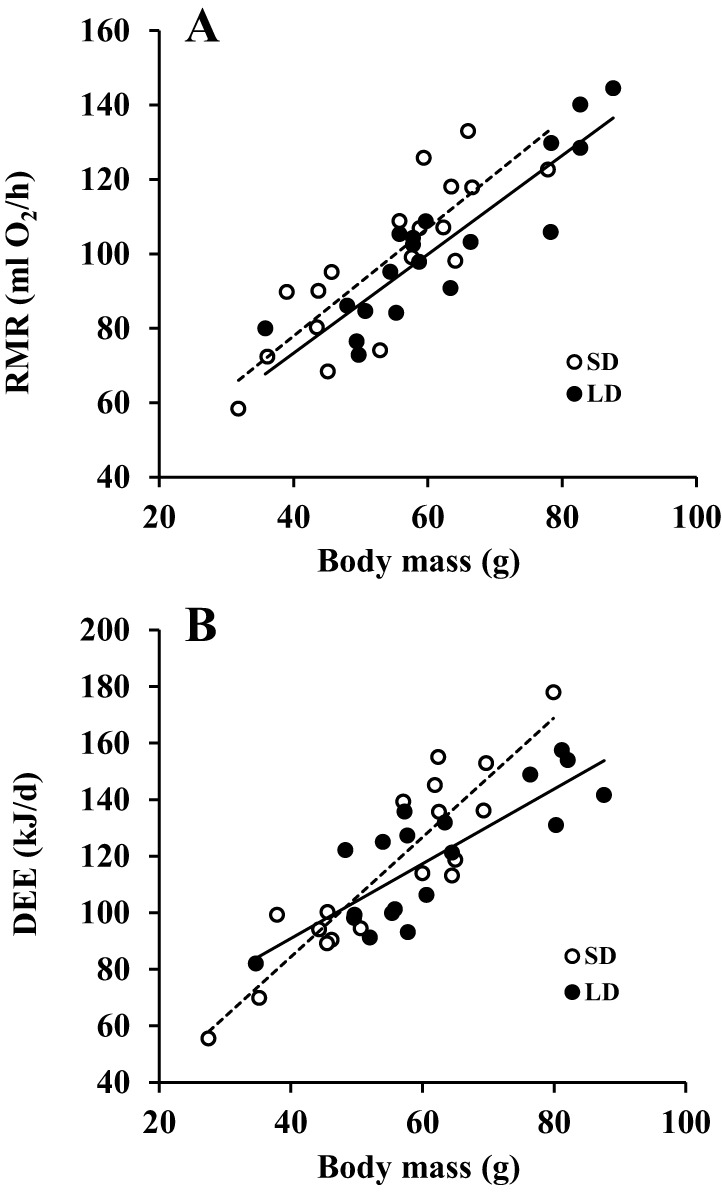
**Effects of photoperiod treatment on energy expenditure of Brandt's voles.** (A) Resting metabolic rate (RMR: oxygen consumption/h) measured by indirect calorimetry. (B) Daily energy expenditure (DEE: kJ/day) measured by doubly-labelled water. White dots are short day exposed animals (*n*=18) and black dots are long day exposed animals (*n*=19). The dashed lines are the fitted regression lines for SD voles and the solid lines are the fitted regression lines for LD exposed voles. There was a trend for resting metabolic rates to be lower in LD animals (*P*=0.075) and a significant reduction in DEE (*P*=0.023).

Resting metabolic rate (RMR) was strongly and positively related to body weight. There was a trend for RMR to be lower in the LD animals when compared with SD animals (*F*_1,34_=3.377, *P*=0.075, ANCOVA; Fig. 3A). Total daily energy expenditure (DEE) was also strongly related to body weight and was significantly lower in the LD animals (*F_1,33_*=5.660, *P*=0.023, ANCOVA; Fig. 3B).

### Glucose and insulin tolerance tests

**Fig. 4.**
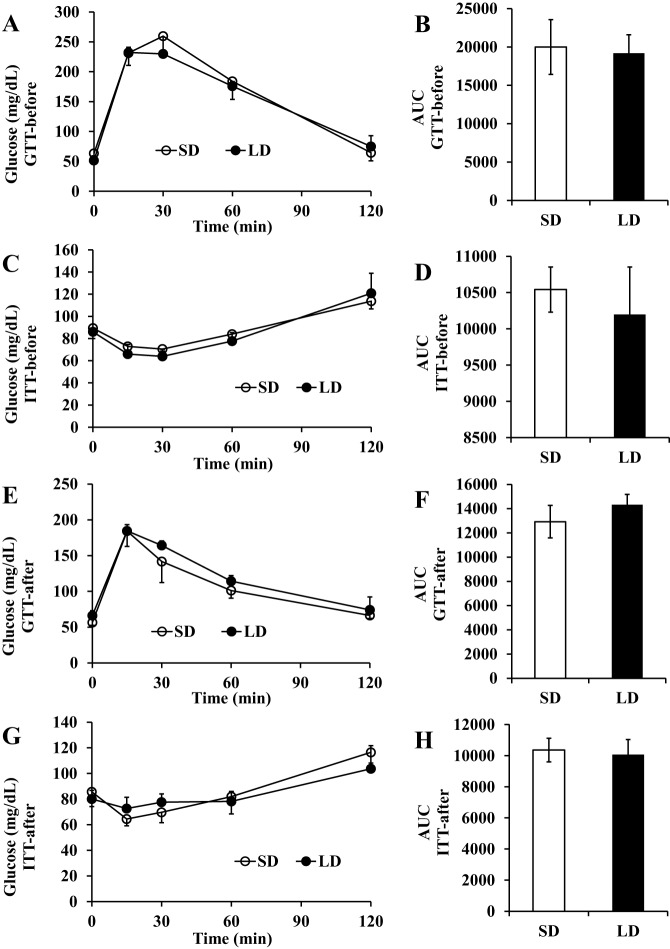
**Effects of photoperiod on glucose tolerance and insulin sensitivity in Brant's voles.** Thirty-seven voles were exposed to short photoperiods. Animals were allocated to experimental groups (SD and LD) and measurements of glucose tolerance (GTT) and insulin sensitivity (ITT) were made on 8-10 animals from each group prior to the voles being exposed to a photoperiod treatment. Following initial measurement the LD voles were exposed to long day photoperiod for 68 days and the SD voles stayed on the short photoperiod. (A-D) Measurements after initial short photoperiod treatment. (A) Time course of glucose in the blood following glucose injection. (B) Area under the curve in A. (C) Time course of glucose in the blood following insulin injection. (D) Area under the curve in C. White dots and bars are short day (SD)- and black dots and bars are long day (LD)-exposed animals. As expected prior to treatment, there were no differences between the groups. (E-H) After 50 days of exposure to treatment the voles were remeasured. (E,G) Time courses of blood glucose following glucose and insulin injection, respectively. (F,H) Areas under the curves in E,G. Given the large fat accumulation in the LD voles it was unexpected that after 50 days there was also no significant effect of photoperiod on either glucose tolerance or insulin sensitivity.

**Fig. 5.**
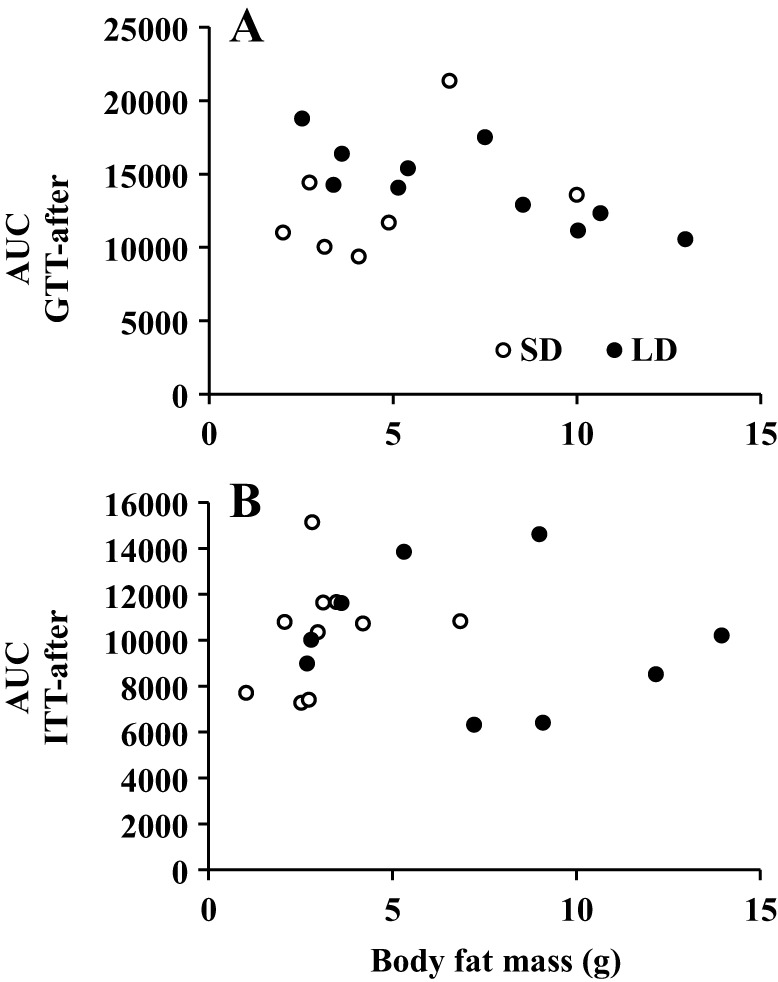
**Effects of body fat content on glucose homeostasis of Brandt's voles following photoperiod manipulation.** Relationships are plotted between individual area under the curve measures from (A) the glucose tolerance test (GTT) and (B) the insulin sensitivity test, for voles exposed to short days (SD: white dots) or long days (LD: black dots) for 60 days. Body fatness and photoperiod had no significant impact on either measurement.

Before the LD exposure, during baseline or after 50 days of LD treatment, both glucose and insulin tolerance tests indicated that blood glucose concentrations of LD animals remained the same as for SD animals, regardless of the divergence in their body weight and fatness (ANOVA *P*>0.05; Fig. 4A,C,E,G). Glucose area under the curve (AUC) from 0-120 min also showed no significant difference between LD treated animals and SD controls (independent sample *t*-tests, *P*>0.05; Fig. 4B,D,F,H). In addition, no correlations were found between body fat mass and GTT or ITT (Pearson's correlation, *P*>0.05; Fig. 5A,B).

### Adipose tissue morphology

**Fig. 6.**
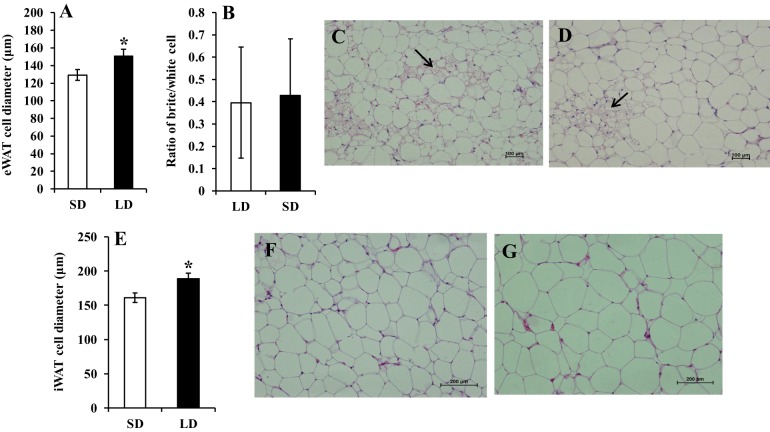
**Effects of photoperiod treatment on WAT cell size of the voles.** (A) Cell diameter of epididymal white adipocyte (eWAT). (B) Ratio of brite/white cells in eWAT. (C) Morphology of eWAT in SD. (D) Morphology of eWAT in LD. (E) Cell diameter of inguinal white adipocyte (iWAT). (F) Morphology of iWAT in SD. (G) Morphology of iWAT in LD. Values are means±s.e.m. (SD, *n*=17; LD, *n*=19). Arrows in C,D point to putative ‘brite’ cells in WAT. *P<0.05 by independent sample *t*-test.

After 70 days of LD treatment, both epididymal and inguinal WAT (eWAT and iWAT, respectively) cell diameter was significantly greater in LD compared with SD animals (eWAT: *t*=2.088, *P*=0.044; iWAT: *t*=2.516, *P*=0.017; Fig. 6). The increase in diameter was equivalent to a cell volume enlarged by 58.4% in eWAT and 60.7% in iWAT when comparing LD animals with SD animals, assuming the shape of the cells approximated a sphere. As the total fat mass increased by about 86% in eWAT and 100.2% in iWAT, the proliferation of adipocytes might also have contributed to the expansion of the tissue mass. We also measured the expression of a number of genes in the eWAT and subcutaneous WAT (scWAT) and the interscapular BAT that are linked to thermogenesis, adipocyte proliferation and fat synthesis. There were no significant differences in *UCP1* expression between LD and SD animals in any of the tissues. In eWAT expression of both *PGC1b* (also known as *Ppargc1b*) *PPARg*, *Cidea*, glucose 6-phosphatase (*G6Pase*, also known as *G6pc*) and fatty acid synthase (*FASN*) were increased under LD treatment (Table 2). Specifically, *FASN* was significantly upregulated (*P*=0.032) and *PGC1b* showed an increase trend (*P*=0.057) in LD animals. These effects were not replicated in the other tissues. Variation in the expression estimates for scWAT was high, perhaps reflecting heterogeneity of this tissue. There were no significant differences in the levels of *PGC1a* (also known as *Ppargc1a*), *PPARg* *Cidea* and *G6Pase* between the two groups in any of the tissues.

**Table 2.**
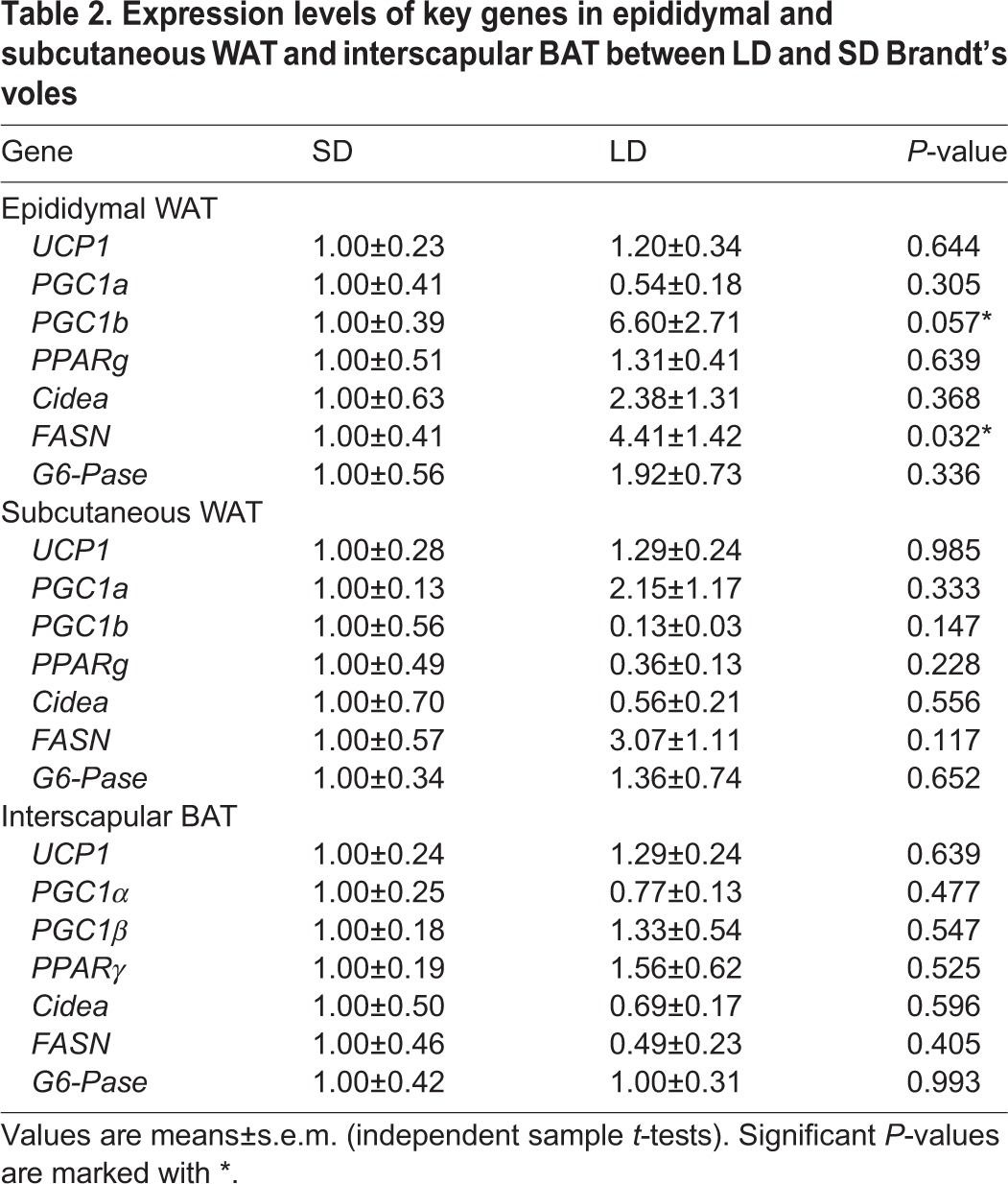
**Expression levels of key genes in epididymal and subcutaneous WAT and interscapular BAT between LD and SD Brandt's voles**

**Fig. 7.**
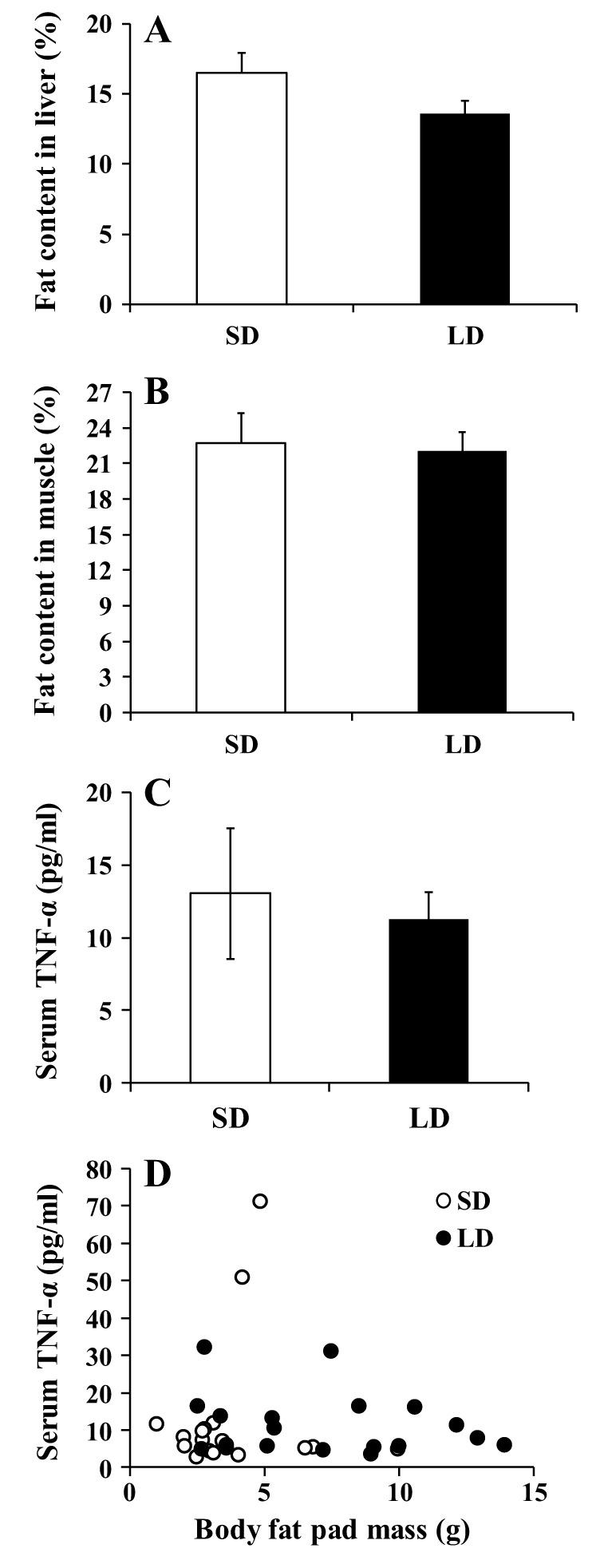
**Effects of photoperiod treatment on liver and muscle fat content and inflammation status of Brandt's voles.** Thirty-seven voles were maintained on short photoperiod, after which 19 of them were exposed to long days and the remaining 18 stayed on short days. (A,B) After 68 days of photoperiod treatment voles were euthanised and measures made of (A) the fat content of the liver and (B) the fat content of skeletal muscle. In both cases there was no significant difference. (C,D) Circulating TNF-α levels were also measured in the serum (C) and these were plotted against the total dissected fat mass (D). There was no significant photoperiod effect or body fatness effect on TNF-α levels. Values in A-C are means±s.e.m.

Despite the obese state in LD animals, fat content of both liver and muscle showed no difference between LD and SD animals (independent sample *t*-tests, *P*>0.05; Fig. 7A,B). Compared with SD voles, LD animals exhibited greater total white fat pad mass. However, this increase in their obesity status was not accompanied by an increase in circulating TNF-α levels (Fig. 7C). No correlation was observed between TNF-α and total body fat mass (Fig. 7D).

## DISCUSSION

When Brandt's voles are exposed to long photoperiods they dramatically increase their body weight. This increase is mostly due to increased size of fat depots when compared with voles maintained in short photoperiods. These changes have been observed previously in other voles (Król et al., 2005; Król and Speakman, 2007) and other small rodents including hamsters (Steinlechner and Heldmaier, 1982) and lemmings (Powell et al., 2002). The photoperiod-induced changes in body weight followed a distinctive two-phase pattern where for about 35 days there was a dramatic increase, followed by a period of stability. In short-tailed field voles we previously showed that during the increase phase the voles were leptin-resistant, and showed a constitutive upregulation of the negative regulator of leptin signalling (SOCS3) that blunts their counter-regulatory response to their expanding body fat (Król and Speakman, 2007). Similar responses in SOCS3 have also been reported in Siberian hamsters (Tups et al., 2004).

Detailed energy budgeting in short-tailed field voles showed that this increased adiposity was mediated neither by gross elevations in food intake, nor reduced resting or physical activity energy expenditure but instead by means of an improvement in digestive efficiency (Król et al., 2005). However, in the present study, we found that the increase in body weight and fatness in Brandt's voles was mediated by decreasing daily energy expenditure, rather than elevated energy intake. This decreased expenditure seemed to be contributed to mostly by a reduction in resting metabolism rather than a decrease in physical activity levels. In lemmings it has also been shown that photoperiod-induced changes in adiposity were associated with differences in energy expenditure that were linked to changes in *UCP1* gene expression in BAT (Powell et al., 2002). In our voles the mass of the BAT was 33% greater in the LD individuals although this difference did not reach significance. In recent years there has been considerable interest in the induction of fat cells with a brown-like appearance in WAT depots, called ‘brite’ or ‘beige’ cells (Nedergaard and Cannon, 2014; Wu et al., 2012). We found no evidence that the ratio of cells putatively identified as ‘brite’ based on their morphology alone, to conventional white adipocytes was altered in the eWAT (Fig. 5) in response to LD photoperiod, consistent with the unaltered levels of *UCP1* in this tissue (Table 2). This suggested that the lowered resting metabolic rate was not linked to such changes. The extent of the decreased energy expenditure was sufficient to explain the increased obesity levels without requiring elevated *de novo* lipogenesis.

Although it has long been recognized that long day photoperiods could lead to obesity in non-hibernating rodents living in the north temperate zone, such as Siberian hamsters (Steinlechner and Heldmaier, 1982), short-tailed field voles (Król et al., 2005) and lemmings (Powell et al., 2002), it is not clear whether metabolic disorders such as impaired glucose homeostasis are associated with the elevated adiposity in these models. Here, we demonstrate that in Brandt's voles photoperiod-induced obesity did not result in glucose intolerance and insulin insensitivity. This absence of an impact of the photoperiod-induced obesity on glucose homeostasis was paralleled by an absence of any increase in the fat content of the liver and skeletal muscle, consistent with the adipose tissue expandability hypothesis (Virtue and Vidal-Puig, 2008). This suggests that photoperiod models of obesity might potentially provide useful insights into the phenomenon of healthy obesity. It will be interesting to know if the other photoperiod-induced obese models of non-hibernating species behave similarly to the Brant's vole studied here.

Contrasting our findings, previous work in hibernating animals suggests they might become insulin resistant when they fatten pre-hibernation, but the data is sparse and conflicting (Martin, 2008; Johnson et al., 2013). However, if it is correct that they do become insulin resistant, this might be because insulin resistance is less likely to be a problem in animals that largely suspend feeding and suppress their metabolic rates (Carey et al., 2003; Storey and Storey, 2004; Heldmaier et al., 2004) for several months when they are most obese, in contrast to voles that remain active and have high levels of energy expenditure year round (Jackson et al., 2001; Speakman et al., 2003; Liu et al., 2003; Li and Wang, 2005; Wu et al., 2009). In this case, contrasting the responses of hibernating and non-hibernating animals might provide valuable insights into the mechanisms involved in healthy obesity. Nevertheless, even hibernators might also show reductions in markers linked to cardiovascular disease and inflammation, suggesting also some adaptive blunting of the links between obesity and its metabolic consequences (Martin, 2008).

The reasons why voles are protected from the negative consequences of obesity are still uncertain but seem in part to be potentially because the animals expanded the size of their existing adipocytes, and increased adipogenesis (based on the difference between the increase in cell volume and total tissue mass increase), allowing them to expand their fat tissue sufficiently to avoid needing to deposit into other tissues such as the liver and muscle. Indeed, we showed that the fat content of these tissues was unrelated to the photoperiod treatment but that adipocytes in the major fat stores expanded in volume by around 60%. The absence of any difference in the markers of adipogenesis (*PGC1a* and *PPARg*) was probably because we measured gene expression levels at the end of the experiment, whereas adipogenesis is probably only transiently elevated during the fat expansion phase. Although this is consistent with the adipose tissue expandability hypothesis, we also noted that there was no increase in circulating TNF-α as a marker of inflammation; hence, avoiding the inflammatory consequences of obesity might be equally important. Defining the mechanism underlying the effect was not our primary aim here and we will further investigate these details in future contributions.

Voles and other photoperiod-responsive small rodents pose several problems for the study of energy balance and the regulation and consequences of adiposity. Because these animals are often wild-derived they are sometimes a challenge to handle and they are generally unacceptable for introduction into SPF facilities where mice are housed; meaning that special housing facilities might be required. However, some species like hamsters are widely used in photoperiod research and can be purchased from SPF suppliers. In reality many pathogens found in voles and hamsters might not transfer to mice so the precautionary exclusion of them from facilities might be too strict. A second problem, however, is that the genomes of these species have not been sequenced. Thus, primers for PCR need to be designed using the mouse or rat genome, which might or might not be successful, and antibodies also might not cross-react with targets of interest. For most physiological measures, however, such as respirometry, body composition analysis, physical activity monitoring, etc., the same procedures that are used in mice and rats can be used without modification. One advantage of the particular species of vole we used is that it is considerably larger than the mouse, and hence provides ample tissue for the performance of assays and histology work. The biggest benefit of using these models, however, is that obesity can be literally induced by the flick of a (light) switch, without modification of the diet – allowing the impacts of obesity to be separated from the impacts of diet.

In summary, photoperiod-induced obesity in the Brandt's vole is achieved by a suppression of energy expenditure under LD conditions, with no change in food intake. The suppressed metabolism was not associated with changes in the ratio of brite to white cells in WAT. Large differences in body fatness in this species were not correlated with changes in glucose intolerance or insulin resistance, suggesting an adaptive decoupling of the obese state from these consequences over evolutionary time. This might be because expanding obesity was also not linked with ectopic fat deposition in either the liver or the skeletal muscle or changes in a marker of inflammation. Photoperiod-induced obesity in non-hibernators such as voles (and perhaps lemmings and hamsters) might provide valuable insights into ‘healthy obesity’.

## MATERIALS AND METHODS

### Animals and experimental design

All animal procedures were carried out with the approval of the Animal Care and Use Committee of the Institute of Genetics and Developmental Biology, Chinese Academy of Sciences. Founder members of the Brandt's vole colony were live-trapped in Inner Mongolia and bred in captivity. The colony is periodically refreshed with individuals from the wild. In this study animals from the colony were maintained at 22±2°C on a long day 16 h:8 h light:dark cycle (lights on at 04:00). Animals were individually housed in plastic cages (30×15×20 cm) with sawdust bedding, food (standard rabbit pellet chow, Beijing HFK Bioscience Co.) and water provided *ad libitum*.

### Energy balance

Male Brandt's voles (*n*=37) were housed in a long day (16L:8D) photoperiod from birth. These voles were transferred into short day (8L:16D) conditions when they were 35 days old and maintained there for 70 days. Voles do not breed in short day conditions so it is not possible to have animals under SD conditions from birth. After 14 days of acclimation to SD, 16 voles were implanted intraperitoneally with a temperature and activity transmitter (15.5 mm×6.5 mm, 1.1 g, Mini Mitter Model G2 E-Mitter) (Chi and Wang, 2011). After SD acclimation for 70 days, the voles were randomly separated into two groups. Animals were randomized using random numbers. We checked after randomization that the body weights were not significantly different between the groups. One group (*n*=18, 8 with transmitters) remained in SD conditions for another 70 days, whereas the other group (*n*=19, 8 with transmitters) were transferred into a LD photoperiod for 70 days. Body weight was measured daily. Researchers weighing the voles were not blinded to the photoperiod treatment as it was impossible to disguise which room was long day and which was short day. The target sample size of 18 per group was established using a power analysis based on previous experience with this vole in response to photoperiod (Zhao and Wang, 2005). Given the known variation in body weight, the sample size provided a power of 0.99 to detect an effect size of the photoperiod treatment of 10 g using a standard two-sample *t*-test (two-tailed, alpha=0.01). As the response to GTT and ITT were previously unknown we could not base the power analysis directly on these traits. We reasoned that if we had a high power to detect the body weight effect then we would be unlikely to fail to detect an effect of GTT and ITT because of insufficient power.

We quantified resting metabolic rate (RMR), daily energy expenditure (DEE) and glucose and insulin tolerance (GTT and ITT, baseline at fourteen days before transfer to LD and sixty two days after LD treatment) of Brandt's voles under the two photoperiods. At the end of photoperiod exposure (day 68), all the animals were fasted for 3-4 h and euthanized by CO_2_ overdose. Once killed all further analyses were performed blind to the original photoperiod treatment. The interscapular BAT, epididymal fat pad, subcutaneous fat pad, pancreas, heart, liver, kidneys and testes were immediately dissected and weighed and stored at −80°C until assayed. Part of the eWAT and iWAT samples were collected and fixed with 4% paraformaldehyde for histological studies. Blood samples were collected, clotted for 1 h and centrifuged at 4°C for 30 min at 1372 ***g***; sera were then collected and stored at −80°C until assayed.

Food intake was measured over 3-day periods centred on days 0, 6, 12, 18, 30, 42, 54 and 66 post-LD exposure. Digestible energy intake was quantified on the same days as food intake measurement. Specifically, voles were presented with a weighed quantity of dry food. Three days later the remaining food and faeces were collected, oven-dried at 60°C to a constant mass and separated manually. Dry matter intake (DMI) was calculated from the difference between the food provided and food remaining. The caloric values of food and faeces were determined by Parr1281 oxygen bomb calorimetry (Parr Instrument USA). Digestible energy intake (DEI) and digestive efficiency were then calculated as follows (Grodzinski and Wunder, 1975; Liu et al., 2002):

DEI (kJ/day)=[dry matter intake (g/day)×food gross energy (kJ/g)]−[dry faeces mass (g/day)×faeces gross energy (kJ/g)]

Digestive efficiency (%)=DEI (kJ/day) / total energy intake (kJ/day)×100.

### Metabolic trials (RMR and DEE)

We measured daily energy expenditure using the doubly labelled water (DLW) technique (Butler et al., 2004) after 48 days of LD exposure. Brandt's voles were weighed (±0.1 g) and injected with ∼0.3 g of water containing enriched ^18^O (31.9 atom %) and ^2^H (19.0 atom %). Syringes were weighed before and after administration (±0.001 g) to calculate the mass of DLW injected. Blood samples were taken after 1 h of isotope equilibration to estimate initial isotope enrichments and were also collected from unlabelled animals to estimate the background isotope enrichments (Król and Speakman, 1999; Visser et al., 2000). Blood samples were immediately heat-sealed into 2×60 μl glass capillaries and stored at room temperature. A final blood sample was taken ∼48 h later to estimate isotope elimination rates (Speakman and Racey, 1988). Taking samples across multiple days minimizes the large between-day variations in DEE estimates (Speakman et al., 1994). We used the intercept method to estimate dilution spaces and estimated the energy expenditure using a single pool model equation (Speakman, 1997, Eqn 7.17), which is appropriate for this size of animal (Speakman and Król, 2005).

Fifty-five days after the different photoperiod exposures started, resting metabolic rate was quantified using indirect calorimetry during the light period (TSE LabMaster, TSE Systems, Germany). Body mass was weighed before each metabolic measurement. RMR was assessed at 30°C, which is in the thermal neutral zone of Brandt's voles (Li and Huang, 1994). Individually housed Brandt's voles were acclimated to the respirometry chamber and both CO_2_ and O_2_ levels were measured every 5 min for 3 h. Animals were not fasted prior to the respirometry run in the chamber. We defined RMR as the average from the 5 min with the least variable and lowest VO_2_ (Duarte et al., 2010).

### Glucose homeostasis

Intra-peritoneal glucose tolerance tests were conducted after fasting overnight. Intra-peritoneal insulin tolerance tests were conducted without fasting. Blood samples were taken by tail veni-puncture for glucose measurements by using a One Touch Ultra Blood Glucose Meter (LifeScan Inc. USA), immediately before, and 15, 30, 60 and 120 min after intra-peritoneal glucose (2 g/kg body mass) or insulin (0.75 IU/kg body mass) administration. The linear trapezoidal rule was used for estimation of area under the curve (AUC).

### Adipose tissue morphology and gene expression

eWAT and iWAT samples were collected and fixed with 4% paraformaldehyde overnight, paraffin-embedded and sectioned to 5 μm in thickness. Three sections of each sample were stained with hematoxylin and eosin (H&E). A Nikon photomicroscope was used for measurement of cell diameters. At 200× magnification the WAT cells were measured at their maximum diameters. About 20 cells of each slide were measured and averaged. For measuring brite to white cell ratios, hematoxylin-labelled cell nuclei were counted and classified; cells with multiple, small droplets were classified as brite cells, cells with single, large droplets were classified as white cells, then brite/white ratios were calculated using brite cell numbers divided by total cell numbers.

### Gene expression of eWAT in LD and SD Brandt's voles

To determine the possible molecular mechanisms regulating energy balance and glucose homeostasis, several thermogenesis-related genes encoding uncoupling protein 1 (UCP1), peroxisome proliferator-activated receptor gamma (PPARγ), peroxisome proliferator-activated receptor gamma coactivator 1-alpha (PGC1α), PGC1β and Cidea, and two glucose-homeostasis-related genes encoding FASN and G6Pase in eWAT were determined using the qPCR method.

### Fat content of liver and muscle

Liver and muscle were collected, weighed and oven-dried at 60°C to constant mass, and then weighed again to obtain the dry mass of tissue. Fat extraction from liver or muscle was performed with a Soxtec Fat Extraction System (Soxtex Avanti 2050, FOSS, Sweden), and then fat content was calculated from the ratio between fat mass and dry mass of tissue.

### Circulating TNF-α

For measurement of TNF-α as an indication of inflammation status we utilized mouse TNF-α ELISA kits (EZMTNFA, Merck Millipore, USA) on the serum samples according to the supplier's instructions.

### Statistical analyses

Data were analysed using SPSS 17.0 software (SPSS Inc., USA). All parameters were tested for normality (Shapiro–Wilks test) and where appropriate log transformed to normalize them before analysis. Group differences between LD and SD groups in white fat pad distributions, organ masses, DEI, RMR and DEE were analysed using analysis of covariance (ANCOVA) with body mass as covariate followed by LSD post-hoc tests (two-tailed, alpha=0.05). Group differences in other parameters (fat-free body mass and AUC) were analysed using independent sample *t*-tests (two-tailed, alpha=0.05). Group differences in body mass, core body temperature, gross activity and digestible energy intake during acclimation were analysed using repeated measures ANOVA (two-tailed, alpha=0.05). Results are presented as means±s.e.m., and *P*<0.05 (two-tailed) was considered to be statistically significant in all tests.

## References

[DMM026070C1] Barroso I., Gurnell M., Crowley V. E. F., Agostini M., Schwabe J. W., Soos M. A., Maslen G. L., Williams T. D., Lewis H., Schafer A. J. et al. (1999). Dominant negative mutations in human PPARγ associated with severe insulin resistance, diabetes mellitus and hypertension. *Nature* 402, 880-883. 10622252 10.1038/47254

[DMM026070C2] Bartness T. J., Demas G. E. and Song C. K. (2002). Seasonal changes in adiposity: the roles of the photoperiod, melatonin and other hormones, and sympathetic nervous system. *Exp. Biol. Med.* 227, 363-376. 10.1177/153537020222700601 12037125

[DMM026070C4] Blüher M. (2010). The distinction of metabolically ‘healthy’ from ‘unhealthy’ obese individuals. *Curr. Opin. Lipidol.* 21, 38-43. 10.1097/MOL.0b013e3283346ccc 19915462

[DMM026070C5] Butler P. J., Green J. A., Boyd I. L. and Speakman J. R. (2004). Measuring metabolic rate in the field: the pros and cons of the doubly-labelled water and heart rate methods. *Funct. Ecol.* 18, 168-183. 10.1111/j.0269-8463.2004.00821.x

[DMM026070C6] Carey H. V., Andrews M. T. and Martin S. L. (2003). Mammalian hibernation: cellular and molecular responses to depressed metabolism and low temperature. *Physiol. Rev.* 83, 1153-1181. 10.1152/physrev.00008.2003 14506303

[DMM026070C7] Chen J.-F., Zhong W.-Q. and Wang D.-H. (2012). Seasonal changes in body mass, energy intake and thermogenesis in Maximowiczi's voles (*Microtus maximowiczii*) from the Inner Mongolian grassland. *J. Comp. Physiol. B* 182, 275-285. 10.1007/s00360-011-0608-9 21874600

[DMM026070C8] Chi Q.-S. and Wang D.-H. (2011). Thermal physiology and energetics in male desert hamsters (*Phodopus roborovskii*) during cold acclimation. *J. Comp. Physiol. B* 181, 91-103. 10.1007/s00360-010-0506-6 20714728

[DMM026070C9] Duarte L. C., Vaanholt L. M., Sinclair R. E., Gamo Y. and Speakman J. R. (2010). Limits to sustained energy intake XII: is the poor relation between RMR and reproductive performance because resting metabolism is not a repeatable trait? *J. Exp. Biol.* 213, 278-287. 10.1242/jeb.037069 20038662

[DMM026070C10] Ebling F. J. P. and Barrett P. (2008). The regulation of seasonal changes in food intake and body weight. *J. Neuroendocrinol.* 20, 827-833. 10.1111/j.1365-2826.2008.01721.x 18601706

[DMM026070C11] Grodzinski W. and Wunder B. (1975). *Ecological Energetics of Small Mammals. Small Mammals: Their Productivity and Population Dynamics*. Cambridge, UK: Cambridge University Press.

[DMM026070C12] Hall K. D., Heymsfield S. B., Kemnitz J. W., Klein S., Schoeller D. A. and Speakman J. R. (2012). Energy balance and its components: implications for body weight regulation. *Am. J. Clin. Nutr.* 95, 989-994. 10.3945/ajcn.112.036350 22434603 PMC3302369

[DMM026070C13] Heldmaier G., Ortman S. and Elvert R. (2004). Natural hypometabolism during hibernation and daily torpor in mammals. *Resp. Physiol. Neurobiol.* 141, 317-329. 10.1016/j.resp.2004.03.014 15288602

[DMM026070C14] Jackson D. M., Trayhurn P. and Speakman J. R. (2001). Associations between energetics and over-winter survival in the short-tailed field vole *Microtus agrestis*. *J. Anim. Ecol.* 70, 633-640. 10.1046/j.1365-2656.2001.00518.x

[DMM026070C15] Johnson R. J., Stenvinkel P., Martin S. L., Jani A., Sánchez-Lozada L. G., Hill J. O. and Lanaspa M. A. (2013). Redefining metabolic syndrome as a fat storage condition based on studies of comparative physiology. *Obesity* 21, 659-664. 10.1002/oby.20026 23401356 PMC3660463

[DMM026070C16] Karelis A. D., Faraj M., Bastard J.-P., St-Pierre D. H., Brochu M., Prud'homme D. and Rabasa-Lhoret R. (2005). The metabolically healthy but obese individual presents a favorable inflammation profile. *J. Clin. Endocr. Metab.* 90, 4145-4150. 10.1210/jc.2005-0482 15855252

[DMM026070C17] Król E. and Speakman J. R. (1999). Isotope dilution spaces of mice injected simultaneously with deuterium, tritium and oxygen-18. *J. Exp. Biol.* 202, 2839-2849. 10504320 10.1242/jeb.202.20.2839

[DMM026070C18] Król E. and Speakman J. R. (2007). Regulation of body mass and adiposity in the field vole, *Microtus agrestis*: a model of leptin resistance. *J. Endocr.* 192, 271-278. 10.1677/JOE-06-0074 17283227

[DMM026070C19] Król E., Redman P., Thomson P. J., Williams R., Mayer C., Mercer J. G. and Speakman J. R. (2005). Effect of photoperiod on body mass, food intake and body composition in the field vole, *Microtus agrestis*. *J. Exp. Biol.* 208, 571-584. 10.1242/jeb.01429 15671345

[DMM026070C20] Li Q. F. and Huang C. X. (1994). Characteristics of the resting metabolic rate of Brandt's voles (*Microtus brandti*). *Acta. Theriologica. Sinica.* 14, 217-220.

[DMM026070C21] Li X.-S. and Wang D.-H. (2005). Regulation of body weight and thermogenesis in seasonally acclimatized Brandt's voles (*Microtus brandti*). *Horm. Behav.* 48, 321-328. 10.1016/j.yhbeh.2005.04.004 15935352

[DMM026070C22] Liu H., Wang D. H. and Wang Z. W. (2002). Maximum metabolizable energy intake in the Mongolian gerbil (*Meriones unguiculatus*). *J. Arid Environ.* 52, 405-411. 10.1007/s00360-005-0022-2

[DMM026070C23] Liu H., Wang D.-H. and Wang Z.-W. (2003). Energy requirements during reproduction in female Brandt's voles (*Microtus brandtii*). *J. Mammal.* 84, 1410-1416. 10.1644/BRG-030

[DMM026070C25] Martin S. L. (2008). Mammalian hibernation: a naturally reversible model for insulin resistance in man? *Diab. Vasc. Dis. Res.* 5, 76-81. 10.3132/dvdr.2008.013 18537093

[DMM026070C28] Nedergaard J. and Cannon B. (2014). The browning of white adipose tissue: some burning issues. *Cell Metab.* 20, 396-407. 10.1016/j.cmet.2014.07.005 25127354

[DMM026070C29] Nichols G. A. and Gomez-Caminero A. (2007). Weight changes following the initiation of new anti-hyperglycaemic therapies. *Diabetes Obes. Metab.* 9, 96-102. 10.1111/j.1463-1326.2006.00580.x 17199724

[DMM026070C30] Ogden C. L., Carroll M. D., Curtin L. R., McDowell M. A., Tabak C. J. and Flegal K. M. (2006). Prevalence of overweight and obesity in the United States, 1999-2004. *JAMA* 295, 1549-1555. 10.1001/jama.295.13.1549 16595758

[DMM026070C31] Powell C. S., Blaylock M. L., Wang R., Hunter H. L., Johanning G. L. and Nagy T. R. (2002). Effects of energy expenditure and UCP 1 on photoperiod-induced weight gain in collared lemmings. *Obes. Res.* 10, 541-550. 10.1038/oby.2002.73 12075603

[DMM026070C32] Savage D. B., Tan G. D., Acerini C. L., Jebb S. A., Agostini M., Gurnell M., Williams R. L., Umpleby A. M., Thomas E. L., Bell J. D. et al. (2003). Human metabolic syndrome resulting from dominant-negative mutations in the nuclear receptor peroxisome proliferator-activated receptor-γ. *Diabetes* 52, 910-917. 10.2337/diabetes.52.4.910 12663460

[DMM026070C33] Scheen A. J. (1999). From obesity to diabetes: why, when and who? *Acta. Clin. Belg.* 55, 9-15. 10.1080/17843286.2000.11754266 10783502

[DMM026070C34] Schwartz M. W. and Porte D. Jr (2005). Diabetes, obesity, and the brain. *Science* 307, 375-379. 10.1126/science.1104344 15662002

[DMM026070C54] Speakman J. R. (1997). *Doubly-labelled Water: Theory and Practice*. London, UK: Chapman and Hall.

[DMM026070C35] Speakman J. R. (2013). Evolutionary perspectives on the obesity epidemic: adaptive, maladaptive, and neutral viewpoints. *Annu. Rev. Nutr.* 33, 289-317. 10.1146/annurev-nutr-071811-150711 23862645

[DMM026070C36] Speakman J. R. and Król E. (2005). Comparison of different approaches for the calculation of energy expenditure using doubly labeled water in a small mammal. *Physiol. Biochem. Zool.* 78, 650-667. 10.1086/430234 15957119

[DMM026070C37] Speakman J. R. and O'Rahilly S. (2012). Fat: an evolving issue. *Dis. Model. Mech.* 5, 569-573. 10.1242/dmm.010553 22915015 PMC3424450

[DMM026070C38] Speakman J. R. and Racey P. A. (1988). Consequences of non-steady state CO_2_ production for accuracy of the doubly labelled water technique: the importance of recapture interval. *Comp. Biochem. Physiol. A Physiol.* 90, 337-340. 10.1016/0300-9629(88)91125-5

[DMM026070C39] Speakman J. R., Racey P. A., Haim A., Webb P. I., Ellison G. T. H. and Skinner J. D. (1994). Inter-and intraindividual variation in daily energy expenditure of the pouched mouse (*Saccostomus campestris*). *Funct. Ecol.* 8, 336-342. 10.2307/2389826

[DMM026070C40] Speakman J. R., Ergon T., Cavagne R., Reid K., Scantlebury D. M. and Lambin X. (2003). Resting and daily energy expenditures of free-living field voles are positively correlated but reflect extrinsic rather than intrinsic effects. *Proc. Natl. Acad. Sci. USA* 100, 14057-14062. 10.1073/pnas.2235671100 14615588 PMC283545

[DMM026070C41] Steinlechner S. and Heldmaier G. (1982). Role of photoperiod and melatonin in seasonal acclimatization of the Djungarian hamster, *Phodopus sungorus*. *Int. J. Biometeorol.* 26, 329-337. 10.1007/BF02219503 7166442

[DMM026070C42] Storey K. B. and Storey J. M. (2004). Metabolic rate depression in animals: transcriptional and translational controls. *Biol. Rev.* 79, 207-233. 10.1017/S1464793103006195 15005178

[DMM026070C55] Tups A., Ellis C., Moar K. M., Logie T. J., Adam C. L., Mercer J. G. and Klingenspor M. (2004). Photoperiodic regulation of leptin sensitivity in the Siberian Hamster, *Phodopus sungorus*, is reflected in arcuate nucleus SOCS-3 (Suppressor of Cytokine Signaling) Gene Expression. *Endocrinology*. 145, 1185-1193. 10.1210/en.2003-1382 14645119

[DMM026070C43] Unger R. H., Clark G. O., Scherer P. E. and Orci L. (2010). Lipid homeostasis, lipotoxicity and the metabolic syndrome. *Biochim. Biophys. Acta Mol. Cell. Biol. Lipids* 1801, 209-214. 10.1016/j.bbalip.2009.10.006 19948243

[DMM026070C45] Virtue S. and Vidal-Puig A. (2008). It's not how fat you are, it's what you do with it that counts. *PLoS Biol.* 6, e237 10.1371/journal.pbio.0060237 18816166 PMC2553843

[DMM026070C46] Visser G. H., Dekinga A., Achterkamp B. and Piersma T. (2000). Ingested water equilibrates isotopically with the body water pool of a shorebird with unrivaled water fluxes. *Am. J. Physiol. Regul. Integr. Comp. Physiol.* 279, R1795-R1804. 11049863 10.1152/ajpregu.2000.279.5.R1795

[DMM026070C47] Warner A., Jethwa P. H., Wyse C. A., I'Anson H., Brameld J. M. and Ebling F. J. P. (2010). Effects of photoperiod on daily locomotor activity, energy expenditure, and feeding behavior in a seasonal mammal. *Am. J. Physiol. Regul. Integr. Comp. Physiol.* 298, R1409-R1416. 10.1152/ajpregu.00279.2009 20200136 PMC2867510

[DMM026070C48] Wu S.-H., Zhang L.-N., Speakman J. R. and Wang D.-H. (2009). Limits to sustained energy intake. XI. A test of the heat dissipation limitation hypothesis in lactating Brandt's voles (*Lasiopodomys brandtii*). *J. Exp. Biol.* 212, 3455-3465. 10.1242/jeb.030338 19837887

[DMM026070C49] Wu J., Boström P., Sparks L. M., Ye L., Choi J. H., Giang A.-H., Khandekar M., Virtanen K. A., Nuutila P., Schaart G. et al. (2012). Beige adipocytes are a distinct type of thermogenic fat cell in mouse and human. *Cell* 150, 366-376. 10.1016/j.cell.2012.05.016 22796012 PMC3402601

[DMM026070C52] Zhao Z.-J. and Wang D.-H. (2005). Short photoperiod enhances thermogenic capacity in Brandt's voles. *Physiol. Behav.* 85, 143-149. 10.1016/j.physbeh.2005.03.014 15924911

[DMM026070C53] Zhao Z.-J. and Wang D.-H. (2006). Short photoperiod influences energy intake and serum leptin level in Brandt's voles (*Microtus brandtii*). *Hormon. Behav.* 49, 463-469. 10.1016/j.yhbeh.2005.10.003 16293255

